# The Effect of Theory-Led Intervention for Knee Osteoarthritis in Older Adults: A Cluster Randomized Trial

**DOI:** 10.1093/geroni/igaa057.1705

**Published:** 2020-12-16

**Authors:** Limin Wang, Hongbo Chen, Han Lu, Jieru Chen, Shaomei Shang

**Affiliations:** Peking University, Beijing, China

## Abstract

Knee osteoarthritis (KOA) is a common joint disease in people over 60 years old. Exercise therapy is one of the most effective non-pharmacological treatments for KOA, but low exercise adherence needs to be improved. This two-arm cluster randomized trial study was to evaluate the effect of the transtheoretical model-lead home exercise intervention (TTM-HEI) program on exercise adherence, KOA symptom (pain intensity and joint stiffness) and knee function (lower limb muscle strength and balance) in Chinese older adults diagnosed with KOA. A total of 189 community-dwelling older adults with KOA (intervention group: n = 103, control group: n = 86) were enrolled from 14 community centers in Beijing, China in 2018. The intervention was a two-stage and 24-week transtheoretical model-based exercise program, and the control group underwent a same length but non-theory-based exercise program. Exercise adherence was measured at weeks 4, 12, 24, 36, and 48 after the program started, KOA symptoms and knee function were measured at baseline, week 24, and week 48. Results showed that the growth rate of exercise adherence in the intervention group increased 2.175 units compared with the control group (unstandardized coefficient of slope on group B2 = 2.175, p < 0.001), and the intervention program maintained participants’ exercise adherence with 5.56 (SD = 1.00) compared with 3.16 (SD = 1.31) in the control group at week 48. In addition, TTM-HEI program showed significant effects on relieving KOA symptoms and improving knee function. This study provided an effective strategy for KOA intervention.

